# A nomogram-based immune-serum scoring system predicts overall survival in patients with lung adenocarcinoma

**DOI:** 10.20892/j.issn.2095-3941.2020.0648

**Published:** 2021-06-15

**Authors:** Qiujuan Huang, Tongyuan Qu, Lisha Qi, Changxu Liu, Yuhong Guo, Qianru Guo, Guangning Li, Yalei Wang, Wenshuai Zhang, Wei Zhao, Danyang Ren, Leina Sun, Shengguang Wang, Bin Meng, Baocun Sun, Bin Zhang, Wenjuan Ma, Wenfeng Cao

**Affiliations:** 1 Department of Pathology, Tianjin Medical University Cancer Institute and Hospital, National Clinical Research Center for Cancer, Key Laboratory of Cancer Prevention and Therapy, Tianjin, Tianjin’s Clinical Research Center for Cancer, Tianjin 300060, China; 2 Department of Pathology, Tianjin Academy of Traditional Chinese Medicine Affiliated Hospital, Tianjin 300120, China; 3 Department of Lung Cancer, Tianjin Lung Cancer Center; 4 Department of Breast Cancer; 5 Department of Breast Imaging, Key Laboratory of Breast Cancer Prevention and Therapy, Tianjin Medical University Cancer Institute and Hospital, National Clinical Research Center for Cancer, Key Laboratory of Cancer Prevention and Therapy, Tianjin, Tianjin’s Clinical Research Center for Cancer, Tianjin 300060, China

**Keywords:** Lung adenocarcinoma, immune microenvironment, nomogram, immunoscore, prognosis

## Abstract

**Objective:**

The immunoscore, which is used to quantify immune infiltrates, has greater relative prognostic value than tumor, node, and metastasis (TNM) stage and might serve as a new system for classification of colorectal cancer. However, a comparable immunoscore for predicting lung adenocarcinoma (LUAD) prognosis is currently lacking.

**Methods:**

We analyzed the expression of 18 immune features by immunohistochemistry in 171 specimens. The relationship of immune marker expression and clinicopathologic factors to the overall survival (OS) was analyzed with the Kaplan-Meier method. A nomogram was developed by using the optimal features selected by least absolute shrinkage and selection operator (LASSO) regression in the training cohort (*n* = 111) and evaluated in the validation cohort (*n* = 60).

**Results:**

The indicators integrated in the nomogram were TNM stage, neuron-specific enolase, carcino-embryonic antigen, CD8_center of tumor (CT)_, CD8_invasive margin (IM)_, FoxP3_CT_, and CD45RO_CT_. The calibration curve showed prominent agreement between the observed 2- and 5-year OS and that predicted by the nomogram. To simplify the nomogram, we developed a new immune-serum scoring system (I-SSS) based on the points awarded for each factor in the nomogram. Our I-SSS was able to stratify same-stage patients into different risk subgroups. The combination of I-SSS and TNM stage had better prognostic value than the TNM stage alone.

**Conclusions:**

Our new I-SSS can accurately and individually predict LUAD prognosis and may be used to supplement prognostication based on the TNM stage.

## Introduction

Lung cancer is the most lethal malignancy worldwide. It has become a major threat to public health^1^. Non-small cell lung carcinoma (NSCLC) accounts for 85% of all primary lung cancer cases, and the most commonly diagnosed pathological type is lung adenocarcinoma (LUAD)^2^. For patients with early-stage LUAD, radical surgical resection remains the preferred treatment. For LUAD, similarly other types of solid tumors, the prognosis is mainly based on tumor, node, and metastasis (TNM) clinical staging after surgery. However, the prognosis varies widely among patients with the same clinical stage. Traditional TNM staging provides limited prognostic information^3^. Therefore, there is an urgent need for a method to improve prediction of patient prognosis and identify high-risk patients among those in similar disease stages.

Accumulating evidence indicates that cancer development is influenced by the host immune system^4,5^. Moreover, an innovative definition of TNM staging, in which T stands for T cells, and M stands for memory cells, has been described^6^. The immunoscore—first proposed by Galon et al. and mainly based on the density of immune cell infiltration in the center of the tumor (CT) and the invasive margin (IM)—has been reported to have prognostic value that may supplement TNM classification in colorectal cancer^7,8^. Thus, the incorporation of immune cells into the new staging system is crucial^1,9^. Unfortunately, an intuitive and effective staging system for predicting LUAD prognosis remains to be developed.

Least absolute shrinkage and selection operator (LASSO) regression has been extended and broadly applied to survival analysis of high-dimensional data^10^. Nomograms are often used as a precise medical tool to predict personalized prognosis by integrating and illustrating statistically significant features^11,12^. Nomograms graphically demonstrate the predicted contributions of different risk factors, and therefore are intuitive and convenient^3,13,14^. In this study, we aimed to screen for key immune indicators and clinicopathological features affecting LUAD prognosis by using LASSO regression in a training cohort. We then used these variables to construct a nomogram that we validated in a validation cohort. Our nomogram-based immune-serum scoring system (I-SSS) was able to further classify patients in the same clinical stage into different risk-based subgroups, thus supplementing prognostication with TNM staging and guiding individualized treatment in clinical settings.

## Materials and methods

### Patients and tissue specimens

In this retrospective study, we selected 171 patients with LUAD who underwent radical surgery at Tianjin Medical University Cancer Institute and Hospital (TMUCIH) between September 2012 and March 2013. This study was approved by TMUCIH. Informed consent was obtained from all individual participants included in the study. The patients did not receive any adjuvant treatment or oncogene screening before surgery. Hematoxylin and eosin-stained tissue sections from all patients were reviewed by 2 pathologists, who then selected the most appropriate tissue sections including the CT and IM regions. Tumor staging was determined according to the American Joint Committee on Cancer (8th edition) criteria. Clinical information for all patients was obtained through review of archived data, and follow-up information was obtained through medical records and telephone interviews. The median follow-up time for the survivors was 68 months (range, 1–72 months). The endpoint of the study was overall survival (OS).

### Immunohistochemistry (IHC)

We selected 9 prognostic immune biomarkers for IHC staining, on the basis of previous research results, including those from pan T cells (CD3), cytotoxic T cells (CD8), B cells (CD20), memory T cells (CD45RO), naive T cells (CD45RA), natural killer cells (CD57), neutrophils (CD66b), macrophages (CD68), and regulatory T cells (FoxP3)^15–22^. IHC for these markers was performed with standard procedures^23,24^. Briefly, 3–4 µm tissue sections were dewaxed in xylene and hydrated through a graded ethanol series. Antigen retrieval was performed at 100 °C in citrate buffer (pH 6.0) for 3 min in all cases, except during CD45RA IHC, for which antigen retrieval was performed at 100 °C in TRIS-EDTA (pH 9.0). After the peroxidase was inactivated with hydrogen peroxide for 20 min, all slides were incubated with the primary antibody overnight at 4 °C. The sections were then successively stained with a broad-spectrum secondary antibody for 1 h at room temperature, treated with 3,3′-diaminobenzidine, and finally counterstained with hematoxylin. Detailed antibody information and staining concentrations are shown in **Supplementary Table S1**.

### Selection of cutoff scores

Two senior pathologists, blinded to clinical information and outcome, independently scored all stained sections. Under a light microscope (model BX51; Olympus), the staining was first evaluated according to overall impression at low magnification (×100), and the 5 most representative areas in the CT and IM region were selected. Next, the densities of the positive cells were scored at high magnification (200×). The stained cells in each area were quantified and expressed as the number of cells per field. The count of each immune marker was the average of the count in the 5 regions. The scoring concordance was approximately 87% between the pathologists. In cases of disagreement, the slides were reviewed collaboratively, and a consensus was reached by the 2 pathologists. For subsequent statistical analyses, each biomarker was recorded as a dichotomous (high *vs.* low) variable according to the optimal cutoff value. For 18 different markers in each tumor region (CT and IM), a corresponding statistically significant correlation was found between the density of immune cells and patient outcomes at a wide range of cutoff values. The optimum cutoff score of the density that produced the “minimum *P*-value” provided the best OS-related stratification^13,25^. The detailed cutoffs and *P*-values are provided in **Table 1**.

**Table 1:** The relationships between 18 immune features and overall survival

Variable	Categories (percentage of positive cells, %)	*P*
CD3_CT_	High (≥10) *vs.* low (<10)	0.256
CD3_IM_	High (≥15) *vs.* low (<15)	0.362
CD8_CT_	High (≥10) *vs.* low (<10)	0.001**
CD8_IM_	High (≥12) *vs.* low (<12)	<0.001**
CD20_CT_	High (≥30) *vs.* low (<30)	0.575
CD20_IM_	High (≥30) *vs.* low (<30)	0.441
CD57_CT_	High (≥1) *vs*. low (<1)	0.140
CD57_IM_	High (≥1) *vs.* low (<1)	0.405
CD66b_CT_	High (≥10) *vs.* low (<10)	0.002**
CD66b_IM_	High (≥10) *vs.* low (<10)	0.197
CD45RA_CT_	High (≥5) *vs.* low (<5)	0.494
CD45RA_IM_	High (≥7) *vs.* low (<7)	0.415
CD45RO_CT_	High (≥25) *vs.* low (<25)	0.014**
CD45RO_IM_	High (≥31) *vs.* low (<31)	0.002**
CD68_CT_	High (≥25) *vs.* low (<25)	0.136
CD68_IM_	High (≥15) *vs.* low (<15)	0.303
FoxP3_CT_	High (≥7) *vs.* low (<7)	<0.001**
FoxP3_IM_	High (≥6) *vs.* low (<6)	0.984

**P < 0.01. CT, center of tumor; IM, invasive margin.

### Statistical analysis

All statistical analyses were performed in SPSS 22.0 (IBM, Chicago, IL, USA). Kaplan-Meier survival and log-rank tests were used to determine the potential correlations of OS with the immune biomarkers and various clinicopathological parameters. Heatmaps and correlation matrices were created with the “pheatmap” package in R (R Core Team. R: A language and environment for statistical computing. R Foundation for Statistical Computing, Vienna, Austria; http://www.R-project.org, 2018). All statistical tests were two-sided. *P* < 0.05 indicated statistical significance.

### Feature selection with LASSO

The 171 patients were divided into training (*n* = 111) and validation (*n* = 60) cohorts in a 65%:35% proportion. LASSO regression was used to further identify predictive features after screening for prognosis-related clinicopathologic characteristics and immune indicators (*P* < 0.05) with the Kaplan-Meier method in the training cohort. LASSO uses both variable selection and regularization to select a subset of variables that minimizes the prediction error of the outcome^26^. Ten-fold cross-validation was performed to assess model classification performance. Feature selection was performed with the “glmnet” package in R.

### Construction and calibration of the prognostic nomogram

On the basis of the aforementioned factors, a multivariate logistic regression model was used to develop a nomogram for predicting LUAD prognosis. In the training cohort, the features obtained through LASSO dimension reduction were included for developing the prognostic nomogram with the R “survival” package. To evaluate the predictive performance of the nomograms, we used the concordance index (C-index), which ranges from 0.5 to 1, with higher values indicating more accurate predictive results. The calibration curves of the nomogram for 2- and 5-year OS were then generated to compare the predicted and observed survival in the validation cohort. Bootstraps with 1,000 resamples were used.

### Risk group stratification based on the nomogram

To further simplify the nomogram, we established a new I-SSS by assigning values to different factors according to the score in the nomogram. By dividing the patients into different risk groups according to the total risk score (from highest to lowest), we determined the cutoff values^3^. Receiver operating characteristic (ROC) analysis was performed to compare the accuracy of I-SSS and TNM stage in predicting prognosis.

## Results

### Clinicopathological characteristics of patients and their relationships to survival

The clinicopathological characteristics of 171 patients and their relationships to OS are summarized in **Table 2**. In total, 71 men and 100 women were enrolled in this study. Most patients were diagnosed at ≥ 50 years of age (87.1%). The preoperative serum tumor markers were as follows: 6.4%, 17.0%, 4.7%, 40.4%, and 28.1% of patients had elevated levels of squamous cell carcinoma antigen (SCC), total prostate-specific antigen (TPSA), neuro-specific enolase (NSE), carcinoembryonic antigen (CEA), and cytokeratin fragment 19 (Cyfra21-1), respectively. The TNM stage distribution was as follows: stage I, 57.3%; stage II, 9.4%; stage III, 21.0%; and stage IV, 12.3%. The histological distribution was as follows: lepidic-predominant, 16.3%; acinar-predominant, 38.6%; papillary-predominant, 9.4%; micropapillary-predominant, 12.9%; solid-predominant, 19.3%; and mucinous-predominant, 3.5%.

**Table 2:** The relationships between clinicopathological features and survival in 171 patients with lung adenocarcinoma

Variable	Total (%)	5-year OS		*P*
		Surviving (%)	Dead (%)	
Gender				0.740
Male	71 (41.5)	41 (57.7)	30 (42.3)	
Female	100 (58.5)	61 (61)	39 (39)	
Age (years)				0.270
<50	22 (12.9)	16 (72.7)	6 (27.3)	
≥50	149 (87.1)	86 (57.7)	63 (42.3)	
History of smoking				0.013*
Absent	102 (59.6)	68 (66.7)	34 (33.3)	
Present	69 (40.4)	34 (49.3)	35 (50.7)	
Hypertension				0.866
Absent	123 (71.9)	74 (60.2)	49 (39.8)	
Present	48 (28.1)	28 (58.3)	20 (41.7)	
Diabetes				0.263
Absent	161 (94.2)	98 (60.9)	63 (39.1)	
Present	10 (5.8)	4 (40)	6 (60)	
Past history				0.671
Absent	160 (93.6)	96 (60.0)	64 (40.0)	
Present	11 (6.4)	6 (54.5)	5 (45.5)	
Family tumor history				0.805
Absent	137 (80.1)	81 (59.1)	56 (40.9)	
Present	34 (19.9)	21 (61.8)	13 (38.2)	
SCC (μg/L)				0.794
≤1.5	160 (93.6)	95 (59.4)	65 (40.6)	
>1.5	11 (6.4)	7 (63.6)	4 (36.4)	
TPSA (U/L)				0.574
≤80	142 (83.0)	86 (60.6)	56 (39.4)	
>80	29 (17.0)	16 (55.2)	13 (44.8)	
NSE (μg/L)				<0.001**
≤15.2	163 (95.3)	102 (62.6)	61 (37.4)	
>15.2	8 (4.7)	0 (0)	8 (100)	
CEA (μg/L)				<0.001**
≤5.0	102 (59.6)	71 (69.6)	31 (30.4)	
>5.0	69 (40.4)	31 (44.9)	38 (55.1)	
Cyfra21-1 (μg/L)				0.020*
≤3.3	123 (71.9)	80 (65.0)	43 (35.0)	
>3.3	48 (28.1)	22 (45.8)	26 (54.2)	
Tumor size (cm)				<0.001**
≤3	122 (71.3)	84 (68.9)	38 (31.1)	
>3	49 (28.7)	18 (36.7)	31 (63.3)	
Lymph node metastasis				<0.001**
Absent	116 (67.8)	87 (75.0)	29 (25.0)	
Present	55 (32.2)	15 (27.3)	40 (72.7)	
Distant metastasis				<0.001**
Absent	78 (45.6)	72 (92.3)	6 (7.7)	
Present	93 (54.4)	30 (32.3)	63 (67.7)		
Histologic style				0.017*
Lepidic predominant	28 (16.3)	24 (85.7)	4 (14.3)	
Acinar predominant	66 (38.6)	38 (57.6)	28 (42.4)	
Papillary predominant	16 (9.4)	10 (62.5)	6 (37.5)	
Micropapillary predominant	22 (12.9)	8 (36.4)	14 (63.6)	
Solid predominant	33 (19.3)	20 (60.6)	13 (39.4)	
Mucinous predominant	6 (3.5)	2 (33.3)	4 (66.7)	
TNM stage				<0.001**
I	98 (57.3)	83 (84.7)	15 (15.3)	
II	16 (9.4)	7 (43.8)	9 (56.2)	
III	36 (21.0)	11 (30.6)	25 (69.4)	
IV	21 (12.3)	1 (4.8)	20 (95.2)	

*P < 0.05; **P < 0.01. SCC, squamous cell carcinoma antigen; TPSA, total prostate-specific antigen; NSE, neuron-specific enolase; CEA, carcino-embryonic antigen; Cyfra21-1, cytokeratin 19 fragments.

Kaplan-Meier analysis showed that the following parameters were significantly associated with OS: history of smoking (*P* = 0.013), NSE level (*P* < 0.001), CEA level (*P* < 0.001), Cyfra21-1 level (*P* = 0.020), tumor size (*P* < 0.001), lymph node metastasis (*P* < 0.001), distant metastasis (*P* < 0.001), histologic style (*P* = 0.017), and TNM stage (*P* < 0.001).

### Expression and correlation analysis of 18 immune cell markers in the CT and IM regions

**Figure 1:**
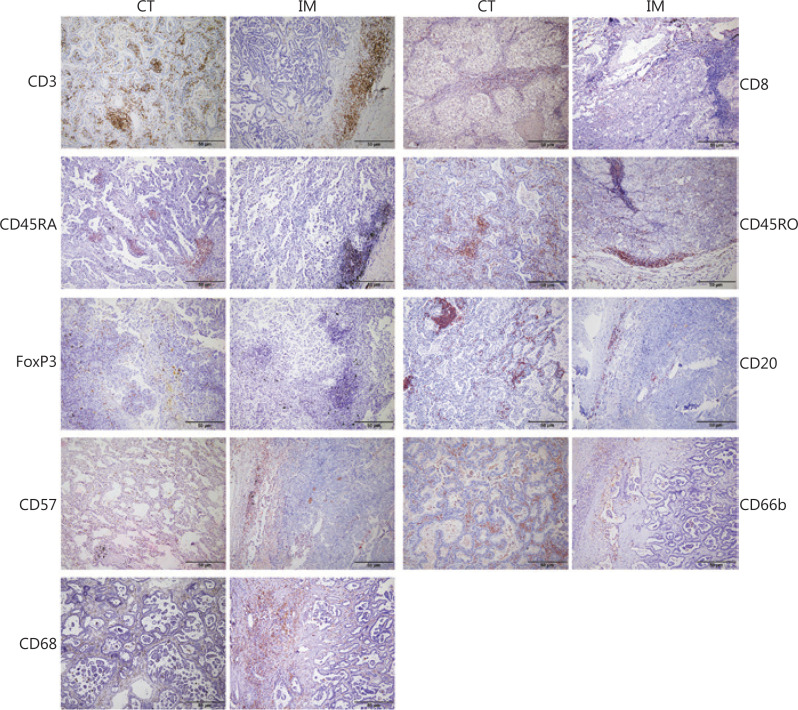
Representative immunohistochemistry images. Staining for 18 immune markers was performed with immunohistochemistry and was detected in the center of the tumor (CT) and the invasive margins (IM) of lung adenocarcinoma (LUAD) specimens (100×).

**Figure 2:**
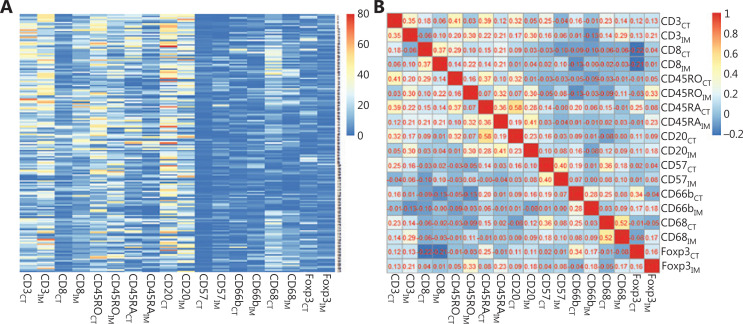
Heatmap and correlation matrix of the expression of 18 immune cells of lung adenocarcinoma (LUAD). (A) Heatmap showing the differential expression of 18 immune cells in LUAD patients, from maximal (red) to minimal (blue) expression levels. (B) Correlation matrix of the correlation of the infiltration of 18 immune cells in LUAD.

We examined the expression of 18 immune cell markers in the CT and IM regions of 171 LUAD specimens with IHC. The staining sites for all indicators were the cell membrane or nucleus (**Figure 1**). **Supplementary Figure S1** shows an example CD3 IHC stained slide with the areas selected for quantification annotated. The heat map (**Figure 2A**) showed distinct immune cell expression profiles in the CT and IM regions of the patients. The densities of CD20_CT_, CD2O_IM_, CD3_CT_, CD3_IM_, CD45RO_CT_, CD45RO_IM_, CD45RA_CT_, and CD8_IM_ were generally high. Meanwhile, the densities of CD8_CT_, CD45RA_IM_, CD68_CT_, CD68_IM_, CD66b_CT_, CD66b_CT_, CD57_CT_, CD57_IM_, FoxP3_CT_, and FoxP3_IM_ were relatively low (**Figure 2A**). In addition, the correlation of the various immune cell markers in LUAD varied from weak to moderate (**Figure 2B**).

### Prognostic effects of immune cell expression on survival

**Figure 3:**
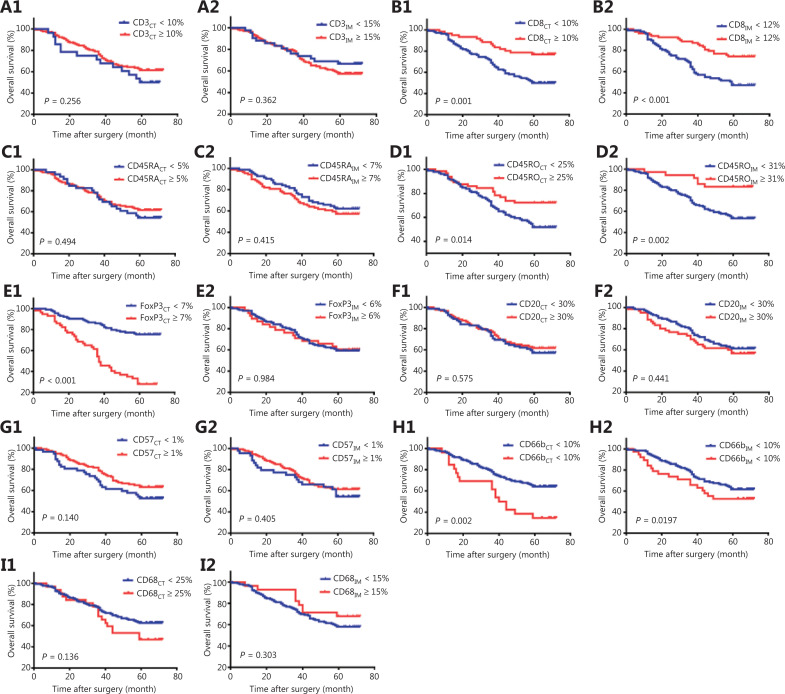
Prognostic value of 18 immune markers in lung adenocarcinoma (LUAD). Kaplan-Meier curves for overall survival (OS), showing that high-density CD8_center of tumor_ (CT) (B1), CD8_invasive margin_ (IM) (B2), CD45RO_CT_ (D1), and CD45RO_IM_ (D2) were associated with longer OS, whereas high-density FoxP3_CT_ (E1) and CD66b_CT_ (H1) were associated with shorter OS. The remaining variables had no significant association with OS (A1, A2, C1, C2, E2, F1, F2, G1, G2, H2, I1, I2).

The cutoff values for different immune makers in the CT and IM regions were obtained with the “minimum *P*-value” method^25^. In Kaplan-Meier analysis, the patients with high densities of CD8_CT_ (*P* = 0.001), CD8_IM_ (*P* < 0.001), CD45RO_CT_ (*P* = 0.014), and CD45RO_IM_ (*P* = 0.002) had significantly better OS than those with low densities of CD8_CT_, CD8_IM_, CD45RO_CT_, and CD45RO_IM_ (**Table 2**, **Figure 3**). Meanwhile, the patients with high densities of CD66b_CT_ (*P* = 0.002) and FoxP3_CT_ (*P* < 0.001) had significantly poorer OS than those with low densities of CD66b_CT_ and FoxP3_CT_ (**Table 2**, **Figure 3**). None of the other immune markers in either region had significant prognostic value (**Table 2**, **Figure 3**).

### Feature selection with LASSO

**Figure 4:**
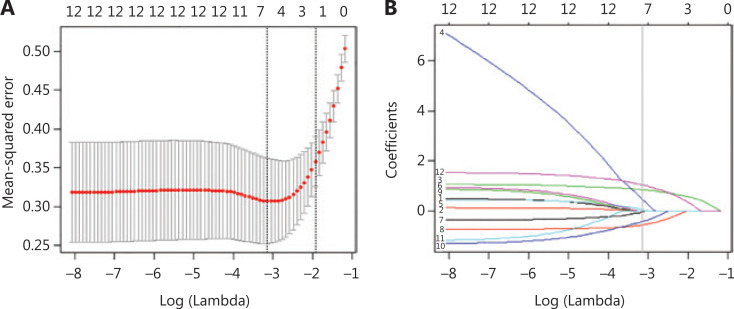
Feature selection using the least absolute shrinkage and selection operator (LASSO). (A) The selection of tuning parameter lambdas in the LASSO model used 10-fold cross-validation *via* minimum criteria. The mean-squared error curve was plotted *vs.* log (lambda). Dotted vertical lines were drawn at the optimal values (log [lambda] −3.142 and −1.933) using the minimum criteria and 1 standard error of the minimum criteria. (B) LASSO coefficient profiles of the 12 features. The vertical line was plotted at the optimal lambda value, which resulted in 7 features with nonzero coefficient were selected.

To comprehensively evaluate the influence of the clinicopathological parameters and the immune markers on prognosis, we selected all significant factors (*P* < 0.05) in the Kaplan-Meier analysis in the training cohort, including 6 of the 17 clinicopathological characteristics and 6 of the 18 immune markers. Because the TNM stage contains tumor size, lymph node metastasis, and distant metastasis, we used only TNM stage in the LASSO screening^13^. The features obtained with LASSO screening included 3 clinicopathological characteristics (TNM stage and preoperative serum NSE and CEA levels) and 4 immune features (CD8_CT_, CD8_IM_, CD45RO_CT_, and FoxP3_CT_) (**Figure 4A** and **4B**, **Supplementary Table S2**).

### Nomogram development for OS and validation

**Figure 5:**
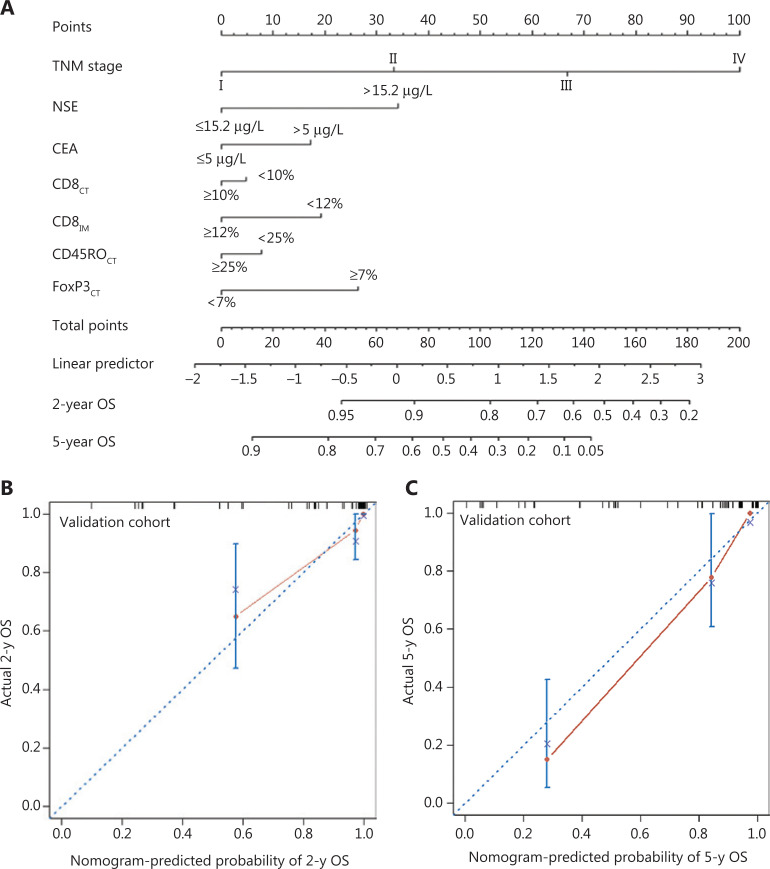
Construction of the prognostic nomogram. (A) The nomogram predicting 2- and 5-year overall survival (OS) of patients with lung adenocarcinoma (LUAD) in the training cohort. (B and C) The calibration curves for predicting 2- (B) and 5-year OS (C) in the validation cohort. The nomogram-predicted probability of survival and actual survival are plotted on the x- and y-axes, respectively. The plot along the 45° line represents a perfect prediction.

Although more prognostic features were selected, the complex interrelationships between variables and the weighted contribution of each factor to tumor formation and development remained unclear. Therefore, a more comprehensive and intuitive model to predict OS was required. Nomograms developed by considering individualized calculations of outcomes on the basis of clinical and pathological features are usually used to predict prognosis. In the training cohort, 7 variables identified with LASSO regression were used to establish a nomogram for OS prediction (**Figure 5A**). The C-index for OS prediction was 0.89, thus indicating very high predictive performance of the model. The calibration curve showed prominent and acceptable agreement between the observed and nomogram-predicted 2- and 5-year OS in the validation cohort (**Figure 5B** and **5C**).

### Performance of the immune-serum scoring system in stratifying patient risk

**Figure 6:**
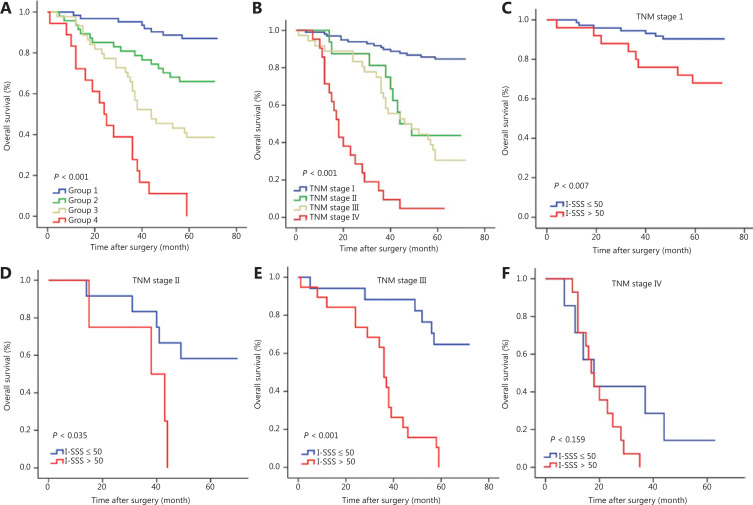
Comparison of the immune-serum scoring system (I-SSS) and tumor, node, and metastasis (TNM) stage in predicting prognosis, and risk group stratification within each TNM stage. (A) The I-SSS was able to classify all patients into 4 subgroups according to cutoff values of 25, 50, and 75. (B) Survival curves grouped according to TNM staging in all patients. (C–F) Risk group stratification within each TNM stage.

To enable more extensive and convenient clinical use, we developed a new I-SSS based on the points awarded for each factor in the nomogram. TNM stages I, II, III, and IV corresponded to 0, 33, 65, and 100 points, respectively. Serum NSE levels >15.2 µg/L corresponded to 34 points. Serum CEA levels >5 µg/L corresponded to 18 points. Low-density CD8_CT_ (<10%), CD8_IM_ (<12%), and CD45RO_CT_ (<25%), and high-density FoxP3_CT_ (≥ 7%) corresponded to 5, 20, 8, and 26 points, respectively. We determined the cutoff value by grouping the patients evenly into 4 subgroups after sorting by total score (score: 0–25, 26–50, 51–75, and >75); each group represented a distinct prognosis (*P* < 0.001); the higher the I-SSS score, the poorer the prognosis (**Figure 6A**). The I-SSS performed better than TNM staging in revealing the differences in prognosis between groups 2 and 3 (**Figure 6A** and **6B**). In patients with the same TNM stage, the independent discrimination ability of the I-SSS was further illustrated. After 50 was used as the cutoff value to group patients, stratification into different risk subgroups resulted in prominent differences in the Kaplan-Meier curves for OS within each TNM stage (**Figure 6C–6F**).

**Figure 7:**
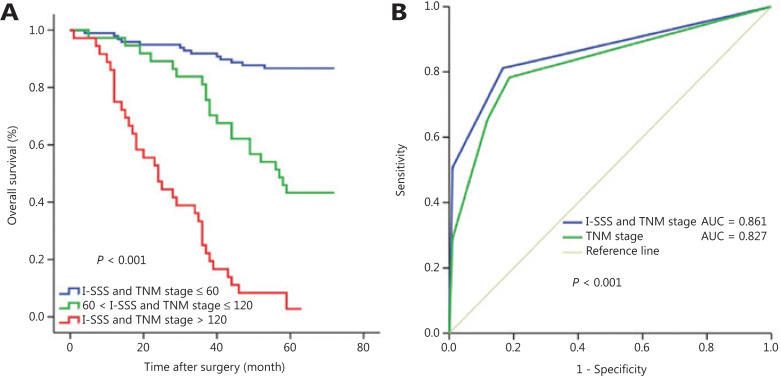
Kaplan-Meier survival analysis of overall survival (OS), based on the combination of immune-serum scoring system (I-SSS) and tumor, node, and metastasis (TNM) stage. (A) The combination of I-SSS and TNM stage could be used to divide all patients into 3 subgroups by using cutoff values of 60 and 120. (B) Receiver operating characteristic (ROC) curve comparing the prognostic accuracy of the combination of I-SSS and TNM stage with that of TNM stage alone.

Furthermore, the combination of I-SSS and TNM stage had a better prognostic value than the TNM stage alone when 60 and 120 were selected as the cutoff values (total score: 0–60, 61–120, >120). Each group had a distinct prognosis (*P* < 0.001) (**Figure 7A**), with a predictive accuracy of OS higher than that of TNM stage (AUC_I-SSS and TNM stage_ = 0.861 *vs.* AUC_TNM stage_ = 0.827) (**Figure 7B**).

## Discussion

In this article, we investigated the associations of the densities and locations of 18 different immune markers with patient survival in LUAD. We also analyzed the effects of various clinicopathological parameters, including tumor markers, on prognosis. In the training cohort, we used LASSO regression to further screen prognostic factors from the results of the Kaplan-Meier analysis, to avoid the problems of multi-collinearity and over-fitting in multiple regression models. Thereafter, we constructed a nomogram by integrating critical prognostic factors for survival. Notably, nomograms are accepted tools for quantifying risk factors, as extensively reported for different cancers. Liang et al.^3^ have built a prognostic nomogram based on general clinical parameters in NSCLC. In addition, Wang et al.^27^ have created a nomogram integrating clinicopathologic features and serum tumor marker levels in NSCLC. In the current study, beyond the basic demographics, the clinicopathologic characteristics and preoperative serum tumor marker levels, and immune infiltrating cells were incorporated into the candidate variables for model building.

Our nomogram ultimately included 3 clinicopathological characteristics (TNM stage, and preoperative serum NSE and CEA levels) and 4 immune features (CD8_CT_, CD8_IM_, FoxP3_CT_, and CD45RO_CT_) that affected LUAD prognosis. Among preoperative serum tumor markers in LUAD, more attention has been focused on CEA, whereas less attention has been focused on NSE. In the present study, in addition to CEA, high levels of NSE were associated with poor prognosis. As described by Li et al.^28^, NSE is a key enzyme in glycolysis that expedites cancer cell replication. Our results indicated that more attention should be paid to NSE in future clinical studies.

CD8 is an important part of the immune microenvironment and plays a crucial role in the anti-tumor immune response^29^. We observed that high CD8^+^ T-cell infiltration in both CT and IM regions was associated with favorable prognosis, similarly to findings from previous studies^21,30^. Donnem et al.^31^ have reported that stromal CD8^+^ T-cell density is an independent prognostic factor for OS, and its prognostic effect increases in different stages in patients with stage I-IIIA NSCLC. FoxP3 is one of the most specific Treg markers, although its effect on prognosis remains ambiguous^23,32,33^. Growing evidence indicates that Tregs play a vital role in promoting cancer by inhibiting the anti-tumor effect of CD8^+^ T-cells and inhibiting host immunity against tumors^23^. Moreover, Wculek et al.^34^ have clarified the key roles played by dendritic cells (DCs) in the initiation and regulation of innate and adaptive immune responses. The roles of Tregs in promoting tumors may be associated with the concomitant absence of DCs, thereby potentiating immunosuppression. In our study, increased FoxP3_CT_ expression was correlated with poor prognosis. This finding was consistent with those of Fu et al.^15^, who have shown that high-density FoxP3 infiltration in the tumor bed in breast cancer is associated with shorter OS. We did not identify a prognostic trend for FoxP3_IM_, possibly because cancer cells in the CT produce chemokines, such as CCL22 and CCR4, thus resulting in lower DC infiltration and recruitment of more FoxP3, and consequently favoring tumor growth^35^. This discovery should be important for guiding immunotherapy for LUAD in the future. CD45RO exerts an antitumor effect, mainly by activating the host immune response^36,37^. In our study, high expression of CD45RO in patients with LUAD had a positive prognostic effect, regardless of whether it was in the CT or IM regions, in agreement with findings from other studies^38^. CD45RO_CT_ rather than CD45RO_IM_ was included the nomogram after LASSO screening, probably because high expression of CD45RO_CT_ had a more pronounced effect than CD45RO_IM_ on prognosis. Recently, Gao et al.^39^ have shown that high density of both CD68_CT_ and CD68_IM_ is associated with decreased survival, and have demonstrated that the macrophage immunoscore-based prognostic nomogram can effectively predict the prognosis of stage I NSCLC patients and enhance the predictive value of the TNM stage system. Unfortunately, we found that neither CD68_CT_ nor CD68_IM_ was associated with prognosis. Further research on the prognostic value of macrophages in lung adenocarcinoma may be warranted. Our results suggested that CD8_CT_, CD8_IM_, FoxP3_CT_, and CD45RO_CT_ might be good candidate immunological markers for establishing a LUAD TNM-immune staging similar to that used in colorectal cancer^1,17,33,40^.

Most importantly, we established a new I-SSS based on the scores for each factor in the nomogram. By stratifying patients with disease into 4 risk groups according to the cutoff values, we separated 171 patients with distinct survival outcomes. Our I-SSS was able to better distinguish the differences in prognosis between group 2 and 3 patients than TNM stage. However, some overlaps in survival curves were observed in TNM stage II and III patients. Furthermore, although patients with the same TNM stage could be stratified into different risk groups with the I-SSS, we did not observe a statistically significant prognostic value in TNM stage IV patients. We believe that the sample size of TNM stage IV was the main contributor to this lack of significance. Patients with low I-SSS and stage I, II, III disease had longer OS than patients with high I-SSS. Therefore, patients with high I-SSS may need more aggressive treatment or intensive follow-up to improve prognosis. In addition, the combination of I-SSS and TNM stage had a better prognostic value than the TNM stage alone (AUC_I-SSS and TNM stage_ = 0.861 *vs.* AUC_TNM stage_ = 0.827), thus indicating that, beyond the TNM stage, the influence of immune cells and tumor markers on LUAD prognosis should not be ignored, and the I-SSS reinforces the prognostic ability of TNM stage^41–43^. These findings suggested that the I-SSS can be used to supplement the prognostic value of TNM staging^44^.

To our knowledge, this study is the first to comprehensively evaluate the effects of the immune microenvironment and clinicopathological features on prognosis, and to develop a nomogram for predicting the survival of patients with LUAD. By using this I-SSS, physicians could provide personalized survival prediction. Moreover, high-risk patients with poor prognosis could be identified and treated with more aggressive therapy or could be followed up more frequently. We note that the existence of anthracotic pigments in lung specimens may mask or confound positively stained cells. We chose to evaluate the IHC staining results by direct microscopic visualization, rather than by using pathological digital software, because microscopy can better distinguish between positive cells and anthracotic pigments^23^. However, this study has some limitations. Although 18 immune features associated with prognosis were selected according to literature reviews and clinical standards, all the features of the immune microenvironment, such as CD4 and CD56, were not represented. Furthermore, this was not a multicenter study; samples from only one hospital were selected. Future studies should examine a larger sample size, specimens from multiple hospitals, and a greater number of immune indicators. A more comprehensive, multi-center, large-scale collaborative study is warranted for further exploration.

## Conclusions

In summary, we developed a new I-SSS to stratify patients with the same TNM stage into different risk subgroups. The combination I-SSS and TNM stage had better prognostic accuracy than that of the TNM stage alone. This comprehensive score system can supplement prognostication based on TNM staging and further guide individualized treatment. Given the importance of personalized medicine and the increasing research on the immune microenvironment, this new I-SSS may provide a crucial foundation for future investigations of immunomodulatory therapies for LUAD.
