# Supplementary material for: A nomogram-based immune-serum scoring system predicts overall survival in patients with lung adenocarcinoma
Source: Cancer Biol Med. 2021 Jun 15;18(2):517–29. doi: 10.20892/j.issn.2095-3941.2020.0648 (PMC8185867; doi:10.20892/j.issn.2095-3941.2020.0648)
Supplement: Supplementary file 1 [file cbm-18-517-s001.pdf]

Supplementary materials

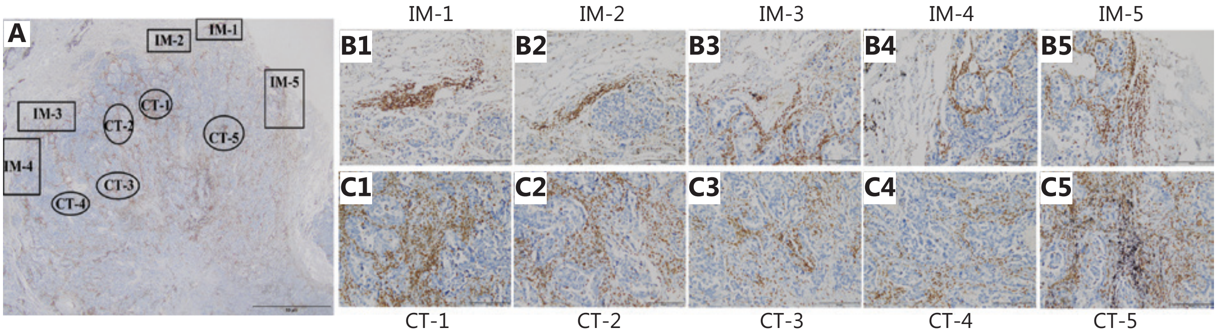

Figure S1 Example CD3 IHC stained slide with the areas selected for quantification annotated. Under a light microscope (model BX51; Olympus), the staining was first evaluated according to overall impression at low magnification (×20) (A), and the 5 most representative areas in the invasive margin (IM) and the center of tumor (CT) region were selected (boxes represent the 5 representative IM areas selected, marked as IM-1, IM-2, IM-3, IM-4, and IM-5; ovals represent the 5 representative CT areas selected, marked as CT-1, CT-2, CT-3, CT-4, and CT-5). The densities of the positive cells were then scored at high magnification (200×) (B1–B5, C1–C5).

Table S1 Antibody information and staining conditions

Markers	Clone number	Manufacturer	Dilution	Antigen retrieval
CD3	SP7	Abcam	1:100	Citrate buffer (pH 6.0) microwave 3 min
CD20	EP459Y	Abcam	1:400	Citrate buffer (pH 6.0) microwave 3 min
CD57	HNK-1/Leu-7	Abcam	1:100	Citrate buffer (pH 6.0) microwave 3 min
CD66b	/	Abcam	1:100	Citrate buffer (pH 6.0) microwave 3 min
CD8	C8/468+C8/144B	Abcam	1:100	Citrate buffer (pH 6.0) microwave 3 min
FoxP3	236A/E7	Abcam	1:100	Citrate buffer (pH 6.0) microwave 3 min
CD45RO	UCHL1	CST	1:400	Citrate buffer (pH 6.0) microwave 3 min
CD68	D4B9C	CST	1:400	Citrate buffer (pH 6.0) microwave 3 min
CD45RA	4KB4	ZSGB-BIO	1:50	pH 9.0 EDTA (pH 9.0) microwave 3 min

CST, Cell Signaling Technology; ZSGB-BIO, Zhongshan Golden Bridge Bio-technology, Beijing; EDTA, ethylenediamine tetraacetic acid.

Table S2 The variables corresponding to each number in LASSO

Number	Variable
1	History of smoking
2	Histologic style
3	TNM stage
4	NSE
5	CEA
6	Cyfra21-1
7	$CD8_{CT}$
8	$CD8_{IM}$
9	$CD66b_{CT}$
10	$CD45RO_{CT}$
11	$CD45RO_{IM}$
12	$FoxP3_{CT}$

NSE, neuron-specific enolase; CEA, carcino-embryonic antigen; Cyfra21-1, cytokeratin 19 fragments; CT, center of tumor; IM, invasive margin.
